# Vitamin D Modulation of the Innate Immune Response to Paediatric Respiratory Pathogens Associated with Acute Lower Respiratory Infections

**DOI:** 10.3390/nu13010276

**Published:** 2021-01-19

**Authors:** Amy S. Bleakley, Paul V. Licciardi, Michael J. Binks

**Affiliations:** 1 Child Health Division, Menzies School of Health Research, Charles Darwin University, Darwin, NT 0810, Australia; 2 Murdoch Children’s Research Institute, Melbourne, VIC 3052, Australia; paul.licciardi@mcri.edu.au

**Keywords:** vitamin D, innate immunity, acute lower respiratory infections, *Streptococcus pneumoniae*, respiratory syncytial virus, influenza virus

## Abstract

Vitamin D is an essential component of immune function and childhood deficiency is associated with an increased risk of acute lower respiratory infections (ALRIs). Globally, the leading childhood respiratory pathogens are *Streptococcus pneumoniae*, respiratory syncytial virus and the influenza virus. There is a growing body of evidence describing the innate immunomodulatory properties of vitamin D during challenge with respiratory pathogens, but recent systematic and unbiased synthesis of data is lacking, and future research directions are unclear. We therefore conducted a systematic PubMed literature search using the terms “vitamin D” and “*Streptococcus pneumoniae*” or “Respiratory Syncytial Virus” or “Influenza”. A priori inclusion criteria restricted the review to in vitro studies investigating the effect of vitamin D metabolites on human innate immune cells (primary, differentiated or immortalised) in response to stimulation with the specified respiratory pathogens. Eleven studies met our criteria. Despite some heterogeneity across pathogens and innate cell types, vitamin D modulated pathogen recognition receptor (PRRs: Toll-like receptor 2 (TLR2), TLR4, TLR7 and nucleotide-binding oligomerisation domain-containing protein 2 (NOD2)) expression; increased antimicrobial peptide expression (LL-37, human neutrophil peptide (HNP) 1-3 and β-defensin); modulated autophagosome production reducing apoptosis; and modulated production of inflammatory cytokines (Interleukin (IL) -1β, tumour necrosis factor-α (TNF-α), interferon-ɣ (IFN-ɣ), IL-12p70, IFN-β, Regulated on Activation, Normal T cell Expressed (RANTES), IL-10) and chemokines (IL-8 and C-X-C motif chemokine ligand 10 (CXCL10)). Differential modulation of PRRs and IL-1β was reported across immune cell types; however, this may be due to the experimental design. None of the studies specifically focused on immune responses in cells derived from children. In summary, vitamin D promotes a balanced immune response, potentially enhancing pathogen sensing and clearance and restricting pathogen induced inflammatory dysregulation. This is likely to be important in controlling both ALRIs and the immunopathology associated with poorer outcomes and progression to chronic lung diseases. Many unknowns remain and further investigation is required to clarify the nuances in vitamin D mediated immune responses by pathogen and immune cell type and to determine whether these in vitro findings translate into enhanced immunity and reduced ALRI in the paediatric clinical setting.

## 1. Introduction

### 1.1. Background

Acute lower respiratory infections (ALRI) are a leading cause of hospitalisation and death in children under-5 globally [1]. The greatest burden occurs in low- and middle- income countries and among socioeconomically disadvantaged populations in high-income countries such as First Nations populations [2]. Early and recurrent ALRIs are also the leading modifiable risk factor for the development of chronic lung diseases which can reduce life expectancy [3]. Slow progress in addressing the social determinants of health drives the need for novel, effective, evidence-based interventions to prevent and/or delay early onset ALRIs.

The most common ALRI presentations in children are acute bronchitis, bronchiolitis, and pneumonia. The key aetiological pathogens include the Gram-positive bacterium *Streptococcus pneumoniae (S. pneumoniae)* [4], respiratory syncytial virus (RSV) [5] and influenza virus [6]. Recently, Severe Acute Respiratory Syndrome Coronavirus 2 (SARS-CoV-2) has caused pandemic infections and is likely to remain a prominent cause of ALRIs moving forward, though mostly in adults [7]. While most of the disease caused by ALRI pathogens is not vaccine preventable, some pathogen specific vaccines are available, such as the pneumococcal conjugate vaccine (PCV) [8] and the inactivated influenza vaccine (IIV) [9]. However, current PCVs only cover up to 13 of the more than 100 *S. pneumoniae* serotypes [10,11] and the IIV is only moderately effective due to annual strain changes in the influenza A virus (IAV) and it is not commonly given to infants [12]. RSV [13] and SARS-CoV-2 [14,15] vaccines are currently under development and may be available in the future. Broader approaches that build infant resilience by supporting optimal immune function are vital.

In this context, vitamin D is an important micronutrient with immunomodulatory properties, yet vitamin D deficiency (<50 nmol/L or 20 ng/mL) is prevalent in many populations around the globe [16,17]. In an extensive analysis spanning 44 countries, 37.3% of the 195 studies examined reported mean values below 50 nmol/L [18]. By region, mean levels were highest in North America and Europe and lowest in the Middle East and Asia. By age, children (especially newborns [18,19,20]) and the elderly (especially those institutionalised [18]) generally exhibited the highest risk of low vitamin D levels. Epidemiological studies have also identified that inadequate vitamin D levels are associated with immunopathological conditions including autoimmune diseases, malignancy and inflammatory diseases [21,22,23,24]. Of interest to the present review, we [19] and others [25] have shown that vitamin D deficiency is linked to an increased risk of ALRI in children. Case-control studies from India [26], Turkey [27] and Bangladesh [25] have found that childhood ALRI hospitalisation was associated with low vitamin D levels. In the Bangladesh study [25], the odds of ALRI doubled for each 10 nmol/L decrease in circulating 25-hydroxyvitamin D3 (25(OH)D_3_). Prospective birth cohort studies in several countries also suggest low cord blood vitamin D is a risk factor for ALRIs. In the Netherlands [28] neonates with 25(OH)D <50 nmol/L (vs >75 nmol/L) had a 6-fold risk of RSV diagnosed ALRI, while in Korea [29] low cord blood 25(OH)D was associated with a 2-fold increased risk of ALRI in the first 6 months of life. Likewise, in northern Australia we discovered that vitamin D deficiency (<50 nmol/L) was common among Aboriginal neonates (44%) and associated with an increased risk of ALRI hospitalisation in the first year of life [19]. While not all studies have found this association, synthesis of data from 12 observational studies [30] suggested that children with 25(OH)D <50nmol/L (vs ≥50 nmol/L) at birth or early infancy had a 3-fold higher risk of ALRI.

There is increasing evidence to show that vitamin D exerts broad regulatory effects on the immune response to infection. Indeed, activation of the vitamin D receptor (VDR) by the active metabolite of vitamin D, 1,25 hydroxy-vitamin D3 (1,25(OH)_2_D_3_), directly and/or indirectly regulates the expression of up to 5% of all the human genes [31,32,33]. Many of the target genes are involved in regulating innate immune function such as expression of pattern recognition receptors (PRRs) [34,35] and various cytokines involved in cellular proliferation, differentiation and degradation [36,37,38]. As vitamin D is intricately linked to innate immune cell function, it is not surprising that deficiency has been associated with inflammatory dysfunction and susceptibility to infection [39,40,41,42]. Furthermore, vitamin D supplementation has been shown to decrease the frequency and severity of respiratory infections [43]. Meta-analysis of individual participant data from 25 randomised controlled trials suggests vitamin D supplementation could reduce the risk of ALRI by over 20% if given daily or weekly during a period of deficiency [43,44].

### 1.2. Vitamin D Metabolism, Signalling, and Function

**Figure 1:**
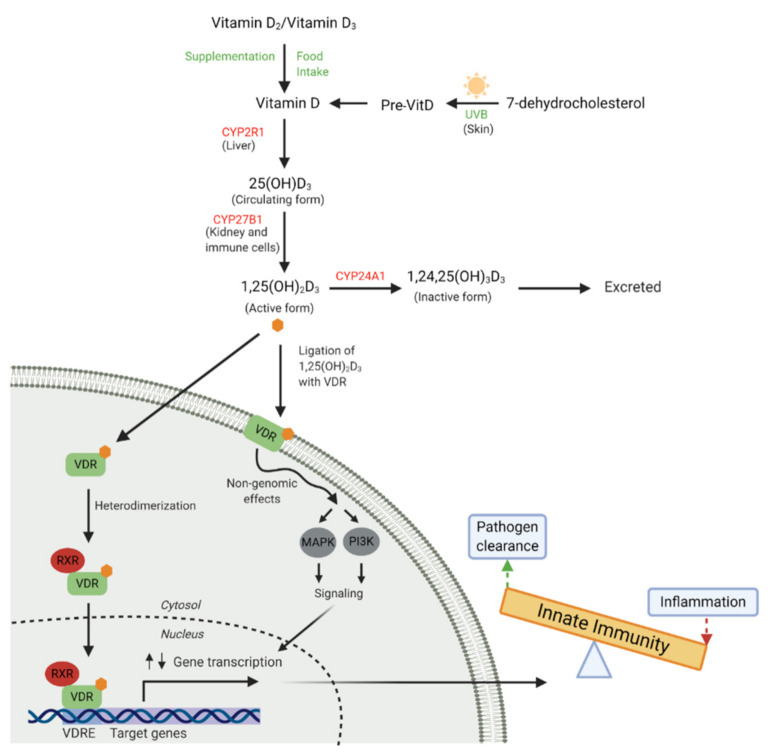
Vitamin D metabolism and receptor binding. Vitamin D is obtained from dietary sources, supplementation or is converted from 7-dehydrocholesterol in the skin by ultraviolet B (UVB) rays. Vitamin D is then converted to the major circulating form, 25(OH)D_3_, by 25-hydroxylase (CYP2R1) in the liver. The active circulating form, 1,25(OH)_2_D_3_, is largely synthesised in the kidney by 1-α-hydroxylase (CYP27B1), but also by many innate immune cells. 1,25(OH)_2_D_3_ ligates with cytosolic or membrane bound vitamin D receptor (VDR) and can also be catabolised by 24-hydroxylase (CYP24A1). VDR ligation results in heterodimerisation with retinoid X receptor (RXR), translocation of this complex to the nucleus and binding to vitamin D response elements (VDRE) in the promotor regions of responsive genes results in up and down-regulation of gene transcription involved in the innate immune response. Non-genomic effects can occur when membrane bound VDR ligation occurs, inducing acute activation of cell signalling pathways (mitogen-activated protein kinase, MAPK and phosphatidylinositol, PI3K). These signalling cascades can alter gene transcription via crosstalk with secondary messengers. (Adapted from Mann et al. [50] and Haussler et al. [51]). (Created with BioRender.com [52]).

Humans synthesise the majority (~90%) of their vitamin D in the skin upon exposure to sunlight with an ultraviolet (UV) index of 3 or higher with the remainder coming from dietary sources [45] (Figure 1). Extreme latitudes, sun avoidance, clothing and deep pigmentation of skin limit epidermal vitamin D production [46], while sunscreens may not limit production as much as previously thought [47]. Cutaneous or dietary sourced vitamin D is hydroxylated in the liver by the enzyme 25-hydroxylase (CYP2R1) to form the major circulating metabolite 25(OH)D_3_; this metabolite reflects total body stores and is considered the best measure of vitamin D status [48]. The active hormonal vitamin D metabolite, 1,25(OH)_2_D_3_, is produced from the circulating 25(OH)D_3_ stores via a second hydroxylation reaction by 1-α-hydroxylase (CYP27B1) in the kidney or locally, as required by cells of other systems. Production of 1,25(OH)_2_D_3_ is tightly regulated by several hormones including parathyroid hormone and other catabolic enzymes in its classic endocrine role of calcium/phosphate metabolism and bone homeostasis [46]. Many human tissues outside of the kidney also have the capacity to synthesise the active vitamin D metabolite (1,25(OH)_2_D_3_) from 25(OH)D_3_, including cells of the innate immune system [49]. This ability to produce 1,25(OH)_2_D_3_ locally at the site of infection or inflammation highlights an important role in the modulation of the immune response.

The cellular actions of vitamin D are exerted upon binding to the VDR, expressed by virtually all cells of the innate immune system, including monocyte/macrophages, respiratory epithelial cells, neutrophils and dendritic cells (DC) [53,54,55,56], making them susceptible to 1,25(OH)_2_D_3_-mediated modulation. Interaction of VDR with its ligand, 1,25(OH)_2_D_3_, induces dimerization with retinoid X receptor (RXR) forming a heterodimeric complex which translocates to the nucleus and binds to vitamin D response elements (VDRE) in vitamin D-responsive genes. Depending on the target gene, other transcription factors may be attracted to the VDR/RXR-complex to ultimately up- or down-regulate gene transcription. [57]. In an alternative non-genomic pathway, 1,25(OH)_2_D_3_ binds to VDR inducing rapid changes in cell signalling pathways, resulting in the activation of one or more secondary messengers, including phosphatidylinositol-3 kinase (P13K) and/or mitogen-activated protein kinase (MAPK) [51,58]. These secondary messengers can engage in crosstalk with genomic responses. Ultimately, the relationship between vitamin D and immune function is complex and remains poorly understood. One of the most well-defined effects of vitamin D on innate immune function is the increased expression of the cathelicidin antimicrobial peptide (CAMP) gene [59,60]. CAMP codes for the human cationic antimicrobial protein 18kDa (hCAP-18) which is subsequently proteolytically cleaved to form the antimicrobial peptide (AMP), LL-37. LL-37 has a broad spectrum of antimicrobial activity against bacteria, fungi and viruses [61].

### 1.3. Hypothesis and Aims

Our overarching hypothesis is that sufficient circulating vitamin D is essential for optimal innate immune responses and protection against ALRIs in early life during a critical period of lung development and adaptive immune maturation, and prior to infant vaccination. While there is an emerging body of evidence from in vitro studies investigating the innate immunomodulatory properties of vitamin D [62,63,64,65] recent systematic and unbiased synthesis of data relating specifically to the leading paediatric ALRIs pathogens are lacking. The aim of this systematic literature review is to describe the current knowledge and knowledge gaps regarding the role of vitamin D metabolites on innate immune cell functions in response to challenge with *S. pneumoniae*, RSV and influenza virus. The evidence reported in this review is intended to update the current knowledge, provide key recommendations for future vitamin D related immunology research and guide the therapeutic potential of vitamin D against ALRIs.

## 2. Methods

**Figure 2:**
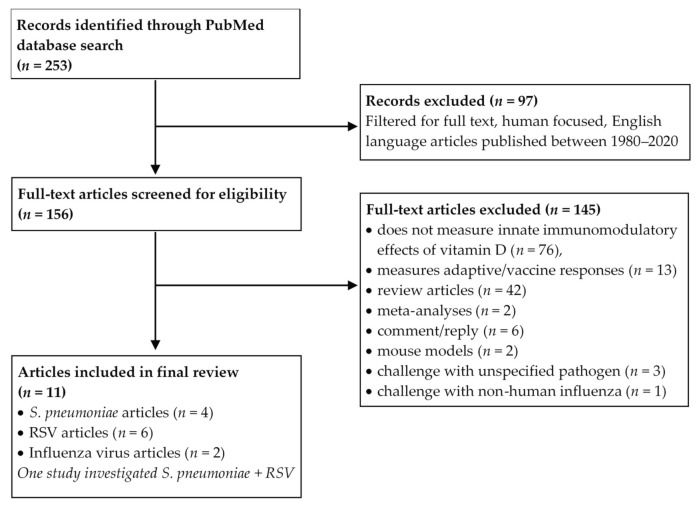
Preferred Reporting Items for Systematic reviews and Meta-Analyses (PRISMA) flow diagram depicting the systematic study selection process. *S. pneumoniae: Streptococcus pneumoniae,* RSV: Respiratory Syncytial Virus.

A PubMed literature search was conducted using Boolean logic with the term “vitamin D” and “*Streptococcus pneumoniae*” or “*Respiratory Syncytial Virus*” or “*Influenza*”. The search was filtered to include only human focused studies published in the English language with full text available, between 1980 and 2020. Our a priori inclusion criteria were in vitro studies encompassing challenge of human innate immune cells with paediatric respiratory pathogens and/or antigens of *S. pneumoniae*, RSV or influenza virus, addressing the innate immunomodulatory effects of vitamin D. Exclusion criteria eliminated studies that did not measure the in vitro immunomodulatory effects of vitamin D in response to pathogen challenge, those focusing on adaptive/vaccine immune responses, review articles, meta-analyses and studies that challenged with pathogens other than those three mentioned above. The study selection process was mapped according to the Preferred Reporting Items for Systematic reviews and Meta-Analyses (PRISMA) guidelines and can be seen in Figure 2. Detailed study characteristics such as cell source, cell type, vitamin D form, dose, duration of exposure, presence of respiratory pathogen stimulus, direction of innate immune function change and pathway were extracted by Amy S. Bleakley (A.S.B) and cross-checked by Michael J. Binks (M.J.B).

## 3. Results

Our final search, conducted on 17th September 2020, retrieved 156 full-text articles after filtering. All articles were carefully assessed by two authors (A.S.B and M.J.B) against the eligibility criteria. Eleven relevant studies were included in the final review (Table 1). Hereafter, the articles and extracted data were organised and presented by challenge pathogen; *S. pneumoniae* (*n* = 4 articles), RSV (*n* = 6 articles), and influenza virus (*n* = 2 articles) (one study investigated *S. pneumoniae* and RSV and is hence included in both sections). None of the studies specifically characterised the responses of immune cells derived from children. A summary of the key findings is presented in Table 1.

### 3.1. Streptococcus Pneumoniae

#### 3.1.1. Background

**Figure 3:**
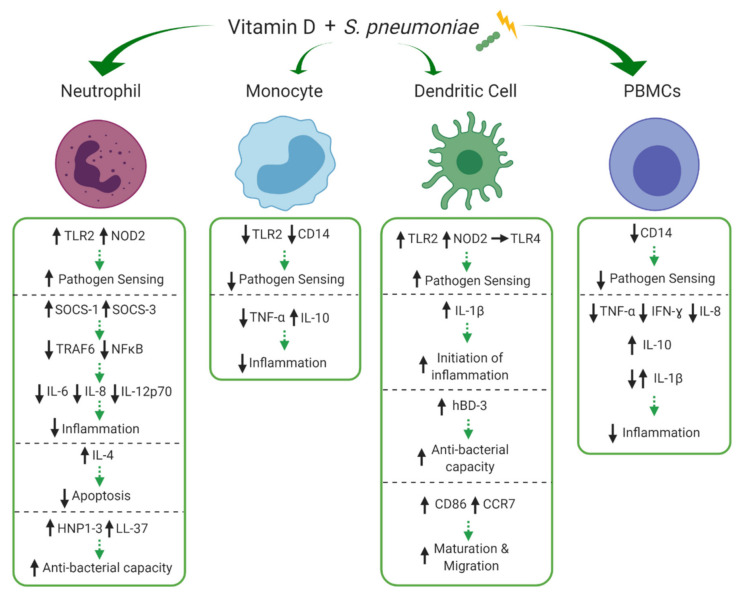
Effects of vitamin D on innate cell subsets in response to *Streptococcus pneumoniae* (*S. pneumoniae)*. Vitamin D differentially modulates expression of pathogen recognition receptors, toll-like receptor 2 (TLR2), TLR4, CD14 and nucleotide-binding oligomerization domain-containing protein 2 (NOD2) on neutrophils, monocytes, dendritic cells (DCs) and peripheral blood mononuclear cells (PBMCs) altering their pathogen sensing capacity. Vitamin D increases production of IL-4 in neutrophils, decreasing apoptosis, whilst also increasing production of suppressor of cytokine signalling 1 (SOCS-1) and SOCS-3, reducing tumour necrosis factor receptor-associated factor 6 (TRAF6) and nuclear factor kappa B (NFκB), overall reducing excessive inflammation (IL-6, IL-8, IL-12p70). Vitamin D modulates TNF-α, IFN-ɣ, IL-8 and IL-10 in monocytes and PBMCs and IL-1β is differentially modulated in PBMCs and DCs, potentially contributing to initiation of the inflammatory process (IL-1β), but overall reducing excessive inflammation. Production of human beta-defensin 3 (hBD-3) within DCs and human neutrophil peptide (HNP1-3) and LL-37 within neutrophils is enhanced by treatment with vitamin D, potentially increasing anti-bacterial capacity. Finally, the maturation and migration (CD86 and C-C chemokine receptor type 7 (CCR7)) of DCs is upregulated by vitamin D, priming these cells for interactions with adaptive immunity. ↑/↓ indicates increase or decrease and → indicates no difference/change to response during immune stimulation with versus without the experimental addition of vitamin D. Green arrows indicate potential net effect. (Created with BioRender.com [52]).

*Streptococcus pneumoniae* (*S. pneumoniae*) is the leading cause of childhood pneumonia worldwide [77]. In 2016, *S. pneumoniae* was responsible for 5.1 million lower respiratory tract infection hospitalisations and 341,029 deaths among children under 5 years across 195 countries [78]. Several clinical studies have linked vitamin D deficiency and pneumonia, showing a significant negative correlation between low serum 1,25(OH)_2_D_3_ levels and pneumonia severity. [79]. The immune response to *S. pneumoniae* in the lungs is multi-faceted involving many aspects of vitamin D dependent innate immunity including mucosal barrier interactions, and optimal functioning of infiltrating and resident immune cells [80]. Our search returned four studies that investigated the influence of vitamin D during pneumococcal challenge. The effects of vitamin D on neutrophils, monocytes, DCs and peripheral blood mononuclear cells (PBMCs) are depicted in Figure 3 and discussed in detail below.

#### 3.1.2. Search Results

Olliver et al. [66] sought to determine the influence of vitamin D on innate DC responses to pneumococcal challenge. In their study, monocyte derived DCs were stimulated with live *S. pneumoniae* strain T4 (encapsulated) or T4R (unencapsulated) or purified *S. pneumoniae* peptidoglycan (PGN) in the presence of 1,25(OH)_2_D_3_. The presence of 1,25(OH)_2_D_3_ during pneumococcal challenge enhanced DC maturation by upregulation of CD86 and increased expression of the migration marker, C-C chemokine receptor 7 (CCR7), which initiates the transit of DCs to draining lymph nodes to engage with the adaptive immune response. Notably, this process was paralleled with reduced cellular uptake of live *S. pneumoniae* strain T4R, indicating that vitamin D-mediated migratory maturation of DCs renders them less phagocytic. Furthermore, in PGN-stimulated DCs 1,25(OH)_2_D_3_ enhanced the gene expression of important PRRs, toll-like receptor (TLR) 2 and nucleotide-binding oligomerization domain-containing protein 2 (NOD2) but did not affect TLR4 gene expression. This demonstrates that vitamin D can elevate the sensing capacity of DCs which could be important for downstream events of PRR activation. Indeed, in this study 1,25(OH)_2_D_3_ also strongly enhanced gene expression of interleukin (IL)-1β and the AMP, human beta defensin 3 (hBD-3), in PGN-stimulated DCs.

Submaranian et al. [68] showed similar effects in neutrophils whereby the active vitamin D metabolite, 1,25(OH)_2_D_3_, induced increased gene and protein expression of PRRs, TLR2 and NOD2, in response to heat killed *S. pneumoniae* strain T4. Further, 1,25(OH)_2_D_3_ treatment of peripheral blood derived primary neutrophils prior to *S. pneumoniae* challenge resulted in significantly reduced levels of the pro-inflammatory cytokines IL-6, IL-8 and IL-12p70 and enhanced production of the anti-inflammatory cytokine IL-4, which reduced the percentage of apoptotic neutrophils. The vitamin D-mediated production of inflammatory cytokines was reduced through the induction of negative regulators of TLR-induced inflammation, suppressor of cytokine signalling (SOCS) proteins, SOCS-1 and SOCS-3 in an IL-4 dependent manner. This resulted in the downregulation of the TLR adaptor protein, tumour necrosis factor receptor-associated factor 6 (TRAF6) and reduction of inhibitor of kappa B alpha (IκBα) phosphorylation which led to suppression of nuclear receptor kappa B (NFκB) nuclear translocation, thereby limiting excessive inflammatory cytokine production. Importantly, neutrophils exposed to 1,25(OH)_2_D_3_ during *S. pneumoniae* infection upregulated expression of the converting enzyme CYP27B1 and the VDR, which in turn enhanced neutrophil killing of *S. pneumoniae* via increased production of the AMPs, LL-37 and human neutrophil peptides 1-3 (HNP1-3).

The innate anti-inflammatory effects of vitamin D have also been shown in healthy PBMCs; which includes monocytes, DCs and natural killer (NK) cells. Hoe et al. [67] showed that pre-treatment (4 h) with vitamin D (both 1,25(OH)_2_D_3_ and 25(OH)D_3_) reduces PBMC production of the pro-inflammatory cytokines tumour necrosis factor alpha (TNF-α), interferon gamma (IFN-ɣ), and IL-1β as well as the chemokine IL-8 in response to challenge (24 h) with heat killed pneumococcal serotype 19F (HK19F). However, in isolated PBMC-derived CD14^+^ monocytes, both forms of vitamin D only reduced the production of TNF-α and unlike in PBMCs, 1,25(OH)_2_D_3_ increased production of the anti-inflammatory cytokine IL-10. This shifted the cytokine ratio (TNF-α:IL-10) towards an anti-inflammatory phenotype in isolated CD14^+^ monocytes. In a subsequent study by the same group [69], longer challenge of PBMCs with pneumococcal whole cell antigen (WCA) was used for the purposes of investigating T helper 17 (Th17) cell responses however some innate cytokines were also measured in these experiments. Results showed that longer 1,25(OH)_2_D_3_ pre-treatment (24 h) of pneumococcal WCA challenged (5 days) PBMCs also increased IL-10 and decreased IFN-ɣ production, while in contrast to their previous study, IL-1β production was increased. Additionally, opposite to the vitamin D-mediated increased TLR2 expression on neutrophils and DCs shown by Subramanian et al. [68] and Olliver et al. [66], respectively, the relative frequency of CD14^+^TLR2^+^ monocytes were reduced by 1,25(OH)_2_D_3_ in response to pneumococcal WCA challenge.

#### 3.1.3. Discussion

From the available data, the net effect of vitamin D metabolites during *S. pneumoniae* challenge was somewhat heterogenous among innate immune cell types. Whilst upregulation of PRRs, TLR2 and NOD2, is increased on neutrophils [68] and DCs [66], CD14^+^TLR2^+^ downregulation is demonstrated on monocytes [69]. The use of different stimulation methods (intact organisms vs cell wall component) among experiments is noteworthy, however it has been suggested by others [81] that vitamin D may prime monocytes to respond less effectively to bacterial cell wall components to reduce the induction of pro-inflammatory mediators via downregulation of TLRs. While the relevance of this in the context of pneumococcal infection remains unclear, these effects may play a role in a negative feedback mechanism, whereby vitamin D prevents excessive TLR activation on monocytes and subsequently reduces inflammation during the course of an infection. The upregulation of PRRs on neutrophils and DCs appears to be synergistic with increased production of AMPs, HNP1-3 and hBD-3, respectively [66,68]. This synergy has been reported in epithelial cells and monocytes treated with vitamin D whereby the NOD2 ligand, muramyl dipeptide (MDP), induces enhanced NFκB-dependent induction of β-defensin 2 expression [82]. Therefore, vitamin D may modulate PRR expression to direct an innate immune response which is effective in clearing *S. pneumoniae* infection and reducing the excessive inflammation associated with dissemination of the bacteria to sterile sites within the host or which facilitates transmission to others. Further studies are required to clarify the differential effects of vitamin D on PRR expression during the early stages of infection.

DCs are highly specialised antigen presenting cells which are critical messengers between the innate and adaptive immune system. This review has highlighted that vitamin D promotes a mature, migratory and non-phagocytic phenotype in DCs in response to pneumococcal challenge [66]. This phenotype is associated with reduced antigen uptake and a shift towards increased antigen presentation and T cell stimulation. This contrasts with much of the previous evidence which shows that 1,25(OH)_2_D_3_ inhibits the differentiation, maturation and immunostimulatory capacity of DCs in response to non-specific TLR activation via lipopolysaccharide (LPS) [83,84,85]. However, while the adaptive immune responses are beyond the scope of this review, it must be mentioned that the mature DC phenotype identified by Olliver et al. [66] was able to modulate the subsequent adaptive T cell response from an inflammatoryTh1/Th17 response toward an anti-inflammatory T cell response [66]. This reduced Th17 response was also observed in PBMCs by Anderson et al. [69], however DC subsets were not specifically investigated. Interestingly, mature DCs display lower levels of VDR and increased expression and activity of CYP27B1 than immature DCs [86]. As such, mature DCs will produce more 1,25(OH)_2_D_3_ yet become relatively insensitive to VDR activity. The excess 1,25(OH)_2_D_3_ may have other non-genomic, paracrine or intracrine functions that are yet to be characterised in this context. The stimulation induced terminal differentiation of DCs may explain why vitamin D induced a mature DC phenotype in the study by Olliver et al. [66], whereby these mature DCs may allow the initiation of a T cell response whilst preventing further DC differentiation and maturation, thereby promoting tolerogenic T cell responses and inhibiting over stimulation of the adaptive immune response. However, it is important to recognise that culture conditions cannot recapitulate the in vivo physiology of DC populations nor vitamin D availability. Prospective research should investigate the vitamin D-mediated phenotype during different stages of DC differentiation and maturation to elucidate the delicate balance between innate and adaptive responses. Modulation of DC differentiation and maturation by vitamin D has been shown to be important during autoimmune diseases [87] and may be important for regulating the inflammatory response caused by *S. pneumoniae* and other respiratory pathogens.

Upon *S. pneumoniae* challenge, vitamin D promotes an anti-inflammatory cytokine profile in monocytes [67] and neutrophils [68] via a reduction in pro-inflammatory cytokines such as TNF-α, IL-6, IL-8, and IL-12p70 and increase in anti-inflammatory cytokines IL-10 and IL-4. An increase in IL-4 subsequently decreases apoptosis of *S. pneumoniae* infected neutrophils [68]. Interestingly, vitamin D increases the production of the pro-inflammatory cytokine, IL-1β, in DCs challenged with pneumococcal PGN (24 h) [66] and in PBMCs challenged with pneumococcal WCA (5 days) [69] however, reduces IL-1β production in PBMCs challenged with HK19F (24 h) [67]. While the concentration of 1,25(OH)_2_D_3_ was consistent among these studies, the length of vitamin D pre-treatment (at time of stimulation vs 24 h vs 4 h), pneumococcal antigen used for stimulation (PGN vs WCA vs HK19F) and length of stimulation (24 h vs 5 days) varied. The differential production of IL-1β between short and long PBMC challenges warrants further investigation. During the early stages of *S. pneumoniae* infection, IL-1β is essential for activation of the epithelium and downstream inflammatory responses [88]. Indeed, an excellent study by Verway et al. [89] demonstrated that vitamin D treated macrophages increase the production of IL-1β during *Mycobacterium tuberculosis (M. tuberculosis)* infection which directly signals to adjacent respiratory epithelial cells increasing their production of AMPs. In the context of ALRI, this mechanism could be highly important to reduce the dissemination of *S. pneumoniae* through the respiratory epithelium. Further work to investigate the vitamin D-inducible production of IL-1β within resident alveolar macrophages (AM) would be highly valuable, though collection of bronchoalveolar lavage samples through bronchoscopy is complex and rarely performed during acute infections. Additionally, whilst vitamin D induced upregulation of AMPs in DCs [66] and neutrophils [68] during pneumococcal challenge in the studies retrieved by our search, this has not yet been reported in monocytes/macrophages. Along with neutrophils, monocytes are recruited to the lungs during ALRI and can differentiate into macrophages. Besides their phagocytic capabilities, macrophages are antigen presenting cells responsible for the initiation of the adaptive immune response, making them important in the defence against many pathogens. Monocytes/macrophages have been shown to promote localised activation of vitamin D in response to *M. tuberculosis* infection, resulting in modulation of LL-37 production [50]. The TLR-mediated induction of LL-37 expression in monocytes is also likely to occur during *S. pneumoniae* infection making vitamin D supplementation a promising approach for treating and/or preventing *S. pneumoniae* infection, particularly among those who are deficient. More research is required to validate vitamin D-mediated production of both IL-1β and LL-37 in response to *S. pneumoniae* infection. It is important to note that Hoe et al. [67] was the only study to report host plasma 25(OH)D_3_ levels, and while still able to observe an effect upon 1,25(OH)_2_D_3_ treatment, baseline vitamin D status may be a confounding factor among other studies utilising adult PBMCs. Studies in known vitamin D-deficient populations may reveal more potent immunomodulatory effects.

Emerging research is beginning to shed light on the vitamin D mediated innate immune cell (neutrophils, monocytes and DCs) responses to *S. pneumoniae.* In addition, the immunomodulatory effects of vitamin D on respiratory epithelial cells in response to *S. pneumoniae* have been evaluated in one interesting study. This study identified a possible polymicrobial mechanism by which treatment with physiological concentrations of 25(OH)D_3_ induced resistance to human rhinovirus (RV) in A549 alveolar epithelial cells and attenuated RV-induced expression of the G-protein coupled receptor, platelet-activating factor receptor (PAFR) [90]. The PAFR is known to mediate adhesion of virulent strains of *S. pneumoniae* to the respiratory epithelium, suggesting that vitamin D may have the capacity to limit post-viral *S. pneumoniae* infection.

In conclusion, the net vitamin D mediated innate immune effect against pneumococcal challenge, though somewhat heterogeneous, comprises differential modulation of PRRs altering pathogen sensing, priming of an anti-inflammatory adaptive immune response via modulation of DC phenotype, increased anti-bacterial capacity via upregulation of AMPs in DCs and neutrophils, and a shift to a predominantly anti-inflammatory cytokine state. Taken together, the findings suggest that vitamin D acts to enhance pneumococcal clearance and reduces excess inflammation associated with immunopathology. Unfortunately, the generalisability of these findings to children remains unclear.

### 3.2. RSV

#### 3.2.1. Background

**Figure 4:**
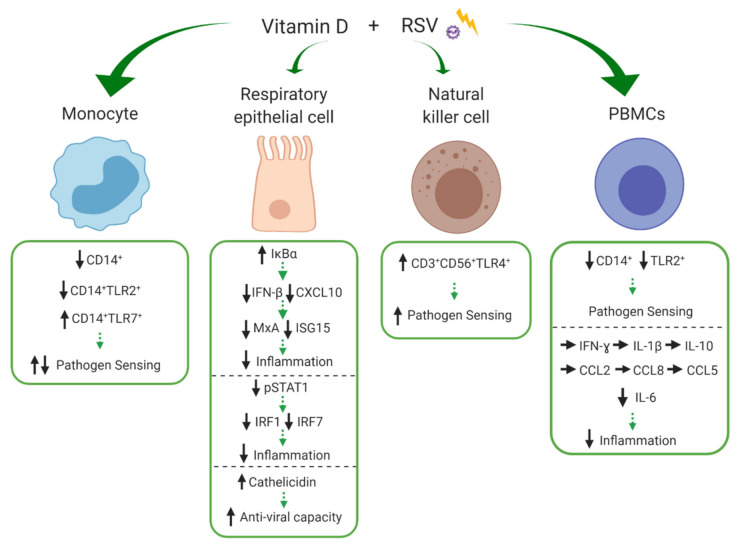
Effects of vitamin D on innate cell subsets in response to respiratory syncytial virus (RSV). Vitamin D differentially modulates expression of pathogen recognition receptors, CD14, toll-like receptor 2 (TLR2), TLR4 and TLR7 on monocytes, natural kills (NK) cells and peripheral blood mononuclear cells (PBMCs) altering their pathogen sensing capacity. Vitamin D increases inhibitor of kappa B alpha (IκBα) and decreases signal transducer and activator of transcription (STAT1) phosphorylation in respiratory epithelial cells, resulting in decreased production of the chemokine; C-X-C motif chemokine ligand 10 (CXCL10) and of antiviral agents; interferon-β (IFN-β), myxovirus resistance protein A (MxA), IFN-stimulated protein of 15kDa (ISG15), IFN regulatory factor 1 (IRF1) and IFR7, overall potentially reducing inflammation. Cathelicidin expression within respiratory epithelial cells is increased by vitamin D, potentially increasing anti-viral capacity. Vitamin D reduces IL-6 in PBMCs but has not impact on IFN-ɣ, IL-1β, IL-10, C-C motif chemokine ligand 2 (CCL2), CCL8, and CCL5. ↑/↓ indicates increase or decrease and → indicates no difference/change to response during immune stimulation with versus without the experimental addition of vitamin D. Green arrows indicate potential net effect. (Created with BioRender.com [52]).

Respiratory syncytial virus is a ubiquitous virus and prominent cause of ALRIs (mostly bronchiolitis) among young children globally. In 2015, there were an estimated 33.1 million episodes of RSV-ALRI, resulting in 3.2 million hospital admissions and 6000 in-hospital deaths in children younger than 5 years [5]. Epidemiological studies have linked vitamin D deficiency to RSV susceptibility, with low 25(OH)D_3_ concentrations associated with increased risk of RSV-associated bronchiolitis in infants [28,91]. RSV primarily infects and replicates within sentinel immune cells of the respiratory epithelium, including respiratory epithelial cells. The virus is recognised by PRRs expressed on innate immune cells, triggering the release of AMPs, chemokines and cytokines, important for the initiation of the inflammatory response to limit viral replication and dissemination [92]. Our search returned six studies that investigated the influence of vitamin D during RSV challenge, the majority of which were experiments in respiratory epithelial cells. The effects of vitamin D on monocytes, respiratory epithelial cells, NK cells and PBMCs are depicted in Figure 4 and discussed in detail below.

#### 3.2.2. Search Results

Early work by Hansdottir et al. (2008) [70] showed that primary human tracheobronchial epithelial (HTBE) cells infected with RSV in the presence of 25(OH)D_3_ were capable of locally upregulating the conversion of 25(OH)D_3_ to 1,25(OH)_2_D_3_ and that this was associated with increased mRNA expression of cathelicidin. Similarly, Telcian et al. [74] showed that pre-treatment of primary human bronchial epithelial cells (HBEC) with 1,25(OH)_2_D_3_ increased RSV-induced cathelicidin mRNA expression in a dose-dependent manner. Together, these data demonstrate that respiratory epithelial cells modulate vitamin D metabolism during RSV infection and increase expression of AMPs, potentially enhancing localised innate anti-viral capacity.

Two studies investigated the vitamin D-mediated PRR-driven cytokine production in PBMCs in response to RSV challenge [69,73]. Fitch et al. [73] found that addition of the active metabolite, 1,25(OH)_2_D_3_, to PBMCs at the time of RSV stimulation had no impact on production of the chemotactic cytokines; C-C motif chemokine ligand 2 (CCL2), CCL8, and CCL5 which control leukocyte chemotaxis or the anti-inflammatory cytokine IL-10. In a similar experiment, Anderson et al. [69] showed that the modulatory effect of 1,25(OH)_2_D_3_ was limited to a reduction in the pro-inflammatory cytokine IL-6, with no effect on other cytokines measured (IFN-ɣ, IL-1β and IL-10). Interestingly, when innate cell subsets were examined [69], 1,25(OH)_2_D_3_ significantly reduced the overall relative frequency of CD14^+^ and TLR2^+^ expression among PBMCs. More specifically, the relative frequency of CD14^+^TLR2^+^ monocytes were reduced, whilst CD14^+^TLR7^+^ monocytes, which mediate viral sensing, and CD56^+^TLR4^+^ NK cells were increased.

Vitamin D also has been shown to modulate important components of signalling cascades that dictate the innate immune response to RSV. A second study by Hansdottir et al. (2010) [71] reported that 1,25(OH)_2_D_3_ modulates NFκB signalling in primary HTBE cells during RSV challenge by increasing mRNA expression of the NFκB inhibitor, IκBα. Importantly, they were able to demonstrate several downstream effects including reduced expression of C-X-C motif chemokine ligand 10 (CXCL10) which acts as a chemoattractant for various immune cells and IFN-β which is an important component of the anti-viral response. In turn, this reduced the induction of IFN-β-stimulated anti-viral proteins; human myxovirus resistance A (MxA) and IFN-stimulated protein of 15kDa (ISG15). Additionally, 1,25(OH)_2_D_3_ decreased RSV-induced levels of signal transducer and activator of transcription (STAT1) protein and its nuclear translocation via decreased STAT1 phosphorylation (pSTAT1). This was demonstrated to be a consequence of NFκB-driven inflammatory suppression (IFN-β). Despite the reduction in various innate anti-viral mediators, the study found no impairment of viral clearance. Similarly, Stoppelenburg et al. [72] investigated the vitamin D mediated immune responses in RSV-infected A549 alveolar epithelial cells. Here, 1,25(OH)_2_D_3_ also reduced the expression of NFκB -driven expression of IFN-β via increased IκBα production and of STAT1-driven antiviral genes (interferon regulatory factor (IRF) 1 and IRF7) via decreased pSTAT1. Furthermore, no differences in viral replication were observed between A549 alveolar epithelial cells expressing the M1 *Fokl* VDR variant (known to be associated with severe bronchiolitis RSV) and those expressing the common M4 VDR variant. However, in M1 *Fokl* VDR-expressing epithelial cells, 1,25(OH)_2_D_3_ had no effect on RSV-induced STAT1 activation and downstream gene expression of IRF1 and IRF7. In summary, these results show that 1,25(OH)_2_D_3_ dampens the innate anti-viral and inflammatory response via NFκB and STAT inhibition, without jeopardizing RSV clearance. Therefore, the vitamin D mediated modulation of anti-viral and inflammatory mediators in response to RSV infection appears to be VDR dependent, with VDR polymorphisms rendering the STAT-mediated immune reactions non-responsive to vitamin D control.

#### 3.2.3. Discussion

Similar to *S. pneumoniae* infection, vitamin D modulates key innate immune responses to RSV. The significance of the differential vitamin D induced modulation of PRRs (TLR2 and TLR7) on monocytes during RSV challenge is unclear [69]. RSV surface proteins can be recognised by TLR2 located on the cell surface and single stranded RNA (ssRNA) can be recognised by TLR7 present on the intracellular endosomal compartment [93]. Activation of these TLRs transmits intracellular signalling which produce cytokines and chemokines important for inflammation and viral clearance. The finding by Anderson et al. [69] that CD3^+^CD56^+^TLR4^+^ NK cell frequency is increased is interesting because TLR4 deficiency can lead to impaired NK cell trafficking to the lungs and significantly impaired cytotoxicity in NK cells in mice during RSV infection [94]. NK cells are important for the recognition of virally infected cells and have strong cytolytic functions [95], therefore the influence of vitamin D on NK cell function could be important in RSV infection. In general, there is limited information regarding the role of PRRs in RSV infection, and PRR subtypes can vary among different cell types eliciting heterogeneous antiviral responses against RSV infection [96]. The results here demonstrate that the effects of vitamin D modulation are likely to be cell type specific. Prospective research is required to validate the differential influence of vitamin D on PRRs among innate immune cells.

In addition to the modulation of PRRs, vitamin D influences key signalling pathways within respiratory epithelial cells involved in the innate immune response to RSV. In this respect, vitamin D increases expression of the NFκB inhibitor, IκBα, and decreases pSTAT1, resulting in decreased production of anti-viral and inflammatory mediators; IFN-β, CXCL10, MxA, ISG15, IRF1 and IRF7 [71,72]. RSV primarily infects respiratory epithelial cells which are potent sources of chemokines and cytokines important for the recruitment and activation of inflammatory cells. While these inflammatory mediators are necessary for an effective host inflammatory response against RSV, uncontrolled inflammation can be deleterious, leading to impaired lung function. Vitamin D (VDR/STAT1-dependent) may be necessary to control RSV-induced inflammation, reducing immunopathology and progression to severe disease. This may be particularly important in infants who are more susceptible to RSV morbidity and mortality.

Importantly, despite the reduced anti-viral responses elicited by vitamin D in response to RSV, viral load and replication is not increased [71,72], even among epithelial cell lines expressing the *Fok*l VDR variant which renders the RSV driven anti-viral inflammatory signalling pathways (STAT1) non-responsive to vitamin D control. This finding indicates that the higher risk of severe RSV bronchiolitis (odds ratio (OR), 2.24; confidence interval (CI), 0.98–5.14) [97,98] among individuals with *Fok*l VDR gene polymorphism (rs2228570; cytosine>thymine (C > T)) is likely related to secondary immunopathology rather than proliferation of the virus itself. Several hypotheses may explain this. In the presence of sufficient vitamin D, the anti-viral response remains entirely functional without being excessive or perhaps the simultaneous induction of cathelicidin [70,74], which has anti-viral activity against enveloped viruses such as RSV [99], counteracts any loss in NFκB-mediated activity, such as IFN-β production. Alternatively, vitamin D may act in a temporal negative feedback loop, where increasing autocrine production of 1,25(OH)_2_D_3_ during an infection dampens the release of potent inflammatory mediators in a concentration and time dependent manner. It has been shown previously that vitamin D can decrease rhinovirus replication and infectivity in HBEC through the induction of cathelicidin [74,100]. The ability to upregulate cathelicidin is one of the key reasons why vitamin D supplementation is attractive as an intervention to protect both infants and adults against RSV infection.

Upon RSV challenge, vitamin D appears to have little net impact on inflammatory cytokine production in PBMCs with effects limited to reduced IL-6 production [69] and no effect on other inflammatory cytokines and chemokines (CCL2, CCL8, CCL5, IFN-ɣ, IL-1β and IL-10) [69,73]. These results contrast with that seen in response to *S. pneumoniae* which displays an anti-inflammatory cytokine profile in the presence of vitamin D. Prospective research should utilise immunophenotyping and intracellular cytokine staining to elucidate the vitamin D-mediated cell-type specific cytokine responses.

In conclusion, vitamin D modulates the inflammatory response to RSV infection by altering PRR expression, reducing the production of proinflammatory cytokines in peripheral innate immune cells and increasing cathelicidin expression in respiratory epithelial cells, potentially increasing anti-viral capacity. Despite the high burden of RSV infection among infants, no studies have specifically investigated the effects of vitamin D on the immune response to RSV in children.

### 3.3. Influenza

#### 3.3.1. Background

Influenza A virus (IAV) infection is a common pathogen identified in children with ALRIs and results in substantial global mortality. In 2008, there was an estimated 20 million episodes of influenza associated ALRI in children under 5 years of age resulting in an estimated 28,000–111,500 deaths worldwide [6]. Observational studies have identified that sufficient levels of vitamin D reduce the chance of developing IAV infection [101] and vitamin D supplementation in humans is associated with reduced incidence and severity during IAV infection [102]. IAV primarily infects respiratory epithelial cells. Within the cells of the immune system, IAV induces apoptosis which is a major contributor to host cell death and tissue damage via stimulation of a pro-inflammatory response [103]. The virus is also able to upregulate the biogenesis of autophagosomes for intracellular survival whilst simultaneously blocking them from fusing with lysosomes. This blockade in autophagy function leads to increased cell stress and apoptosis. Autophagy and apoptosis are two distinct self-destructive cellular processes which control the turnover of cytoplasmic organelles and entire cells, respectively [104]. Our search returned only two studies that investigated the influence of vitamin D during IAV challenge.

#### 3.3.2. Search Results

In 2013, Khare et al. [75] showed that 1,25(OH)_2_D_3_ treatment of A549 alveolar epithelial cells pre- and post-H1N1 (IAV) infection significantly reduces the IAV-induced levels of IL-6, TNF-α, IL-1β, RANTES (Regulated on Activation, Normal T cell Expressed and Secreted) and IL-8. Interestingly, gene expression of IFN-β and ISG15 was significantly reduced by 1,25(OH)_2_D_3_ pre-treatment, however, 1,25(OH)_2_D_3_ treatment post-IAV infection significantly increased both IFN-β and ISG15. Furthermore, treatment with 1,25(OH)_2_D_3_ pre- and post-H1N1 infection reduced autophagy measured by beclin-1 expression and significantly decreased apoptosis, measured by Sub G1 peak analysis, to constitutive levels seen in uninfected control cells. These results did not explain why viral levels and respiratory epithelial cell viability remained unchanged. However, a significant decrease in influenza M protein mRNA levels was observed, indicating reduced viral replication. Though this study identified that vitamin D could modulate the interplay between autophagy and apoptosis, more recent experiments have shed light on the molecular mechanisms by which 1,25(OH)_2_D_3_ regulates these two processes. Godbole et al. [76] demonstrated restoration of autophagy (inhibited by H1N1) marked by increased levels of the autophagy markers microtubule-associated protein light chain 3-Ⅱ (LC3B-Ⅱ) and p62 which represent the levels of autophagosome formation and degradation, respectively. In addition, levels of Syntaxin-17 (STX17) and V-type proton ATPase subunit (ATP6VOA2) which regulate vesicular fusion and lysosomal activity were restored to control levels in the presence of 1,25(OH)_2_D_3._ This was accompanied by reversal of H1N1 induced apoptosis in A549 alveolar epithelial cells, marked by decreased levels of the apoptotic marker Cleaved Caspase-3.

#### 3.3.3. Discussion

The direct molecular basis behind the anti-IAV action of vitamin D is currently not well understood. Vitamin D reduces the pro-inflammatory cytokine prolife produced by IAV infected respiratory epithelial cells [75]. However, the finding that vitamin D treatment post H1N1 infection significantly increases both IFN-β and ISG15 is very interesting and suggests that vitamin D may regulate the inflammatory response in a time-dependent manner. Unfortunately, neither the *S. pneumoniae* nor RSV studies evaluated post infection addition of vitamin D precluding direct contrast in this regard. Though the post-infection activity appears opposite to that observed with vitamin D pre-treatment in both RSV and IAV infection [71,72,75], 1,25(OH)_2_D_3_ might be tightly regulated and less available during this scenario in vivo. Nevertheless, adequate vitamin D present pre- and post-IAV infection suppresses a range of inflammatory mediators which may reduce tissue injury and immunopathology. Investigation into the modulation of intracellular pathways responsible for suppression of cytokines/chemokines within IAV-infected respiratory epithelial cells would be worthwhile to fully elucidate the anti-viral mechanisms modulated by vitamin D.

Importantly, the pro-autophagic actions of vitamin D have been proposed as a possible explanation for the observed anti-IAV activity of vitamin D in humans. Restoration of autophagy during IAV infection potentially limits viral induced cellular injury via reduced apoptosis [75,76]. The mechanisms of autophagy during IAV infection are dynamic and complex and yet to be clearly characterised. Therefore, the results from the studies identified in this review [75,76] should be interpreted with caution since they used different parameters to measure autophagic flux and did not comprehensively address the mechanistic action of vitamin D on autophagy. Several approaches can be used to measure autophagic flux and advancements in autophagy measuring techniques, such as immunofluorescence, may aid the efforts to elucidate the effects of vitamin D on autophagy during IAV infection [105]. Interestingly, vitamin D has been shown to induce anti-mycobacterial activity against *M. tuberculosis* in monocytes/macrophages by increasing autophagy in a cathelicidin-dependent manner [106]. Cathelicidin has been shown to have anti-viral activity against IAV [107] and could be another mechanism by which vitamin D exerts anti-IAV effects.

To summarise, IAV is known to undermine the autophagic processes in respiratory epithelial cells, inducing apoptosis and tissue damage. The studies reviewed here suggest that vitamin D has pleiotropic effects during IAV infection, modulating the production of pro-inflammatory cytokines and anti-viral mediators and restoring IAV inhibited autophagy, thereby limiting cellular apoptosis. As such, vitamin D may be important to limit IAV induced pathology.

## 4. Summary of Findings

### 4.1. S. pneumoniae

- Three of four studies reported modulation of PRRs (TLR2, TLR4 and NOD2) with differential effect across innate cell types (DCs, neutrophils and monocytes), overall, potentially increasing pathogen sensing.
- Two of four studies measured AMP production, hBD-3 in DCs and HNP1-3 and LL-37 in neutrophils, consistently showing increased production and directly effecting bacterial clearance.
- One of four studies reported reduced apoptosis (neutrophils) via increased IL-4 production.
- One of four studies investigated DC phenotype reporting a mature, migratory and non-phagocytic phenotype.
- Two of four studies measured inflammatory cytokines and chemokines (TNF-α, IFN-ɣ, IL-1β, IL-8, IL-10, IL-12p70) displaying a predominantly anti-inflammatory profile with the exception of IL-1β which was differentially modulated by vitamin D across cell types (DCs and PBMCs), however this may be due to experimental design.
- One of four studies investigated intracellular signaling molecules (SOCS1, SOCS3, TRAF6 and NFκB) which ultimately controls the excessive production of pro-inflammatory cytokines.

### 4.2. RSV

- One of six studies reported modulation of PRRs (TLR2, TLR4 and TLR7) with differential effects across innate cell types (monocytes and NK cells).
- Two of six studies measured AMP expression (cathelicidin) in respiratory epithelial cells, consistently showing increased expression potentially increasing anti-viral capacity.
- Two of six studies measured inflammatory cytokines and chemokines (CCL2, CCL8, CCL5, CXCL10, IL-6, IL-10, IFN-ɣ, IL-1β and IL-10) with effects limited to reduced IL-6 production in PBMCs.
- Two of six studies measured anti-viral mediators (IFN-β, MxA, ISG15, IRF1 and IRF7) in respiratory epithelial cells consistently showing reduction, despite no change in viral load or replication.
- Two of six studies investigated intracellular signaling cascades, both showing increased IκBα and decreased pSTAT1 to be associated with the reduction in anti-viral mediators.

### 4.3. Influenza

- Two of two studies measured autophagy showing differential modulation but overall downstream reduction in apoptosis via increased auto-phagolysosome fusion in respiratory epithelial cells.
- One of two studies measured inflammatory cytokines (IL-6, TNF-α, IL-1β, RANTES and IL-8) and anti-viral mediators (IFN-β and ISG15), overall reducing inflammation but differentially modulating anti-viral agents in the presence of vitamin D pre- and post-infection.

## 5. Conclusions

In this systematic literature review, we describe the current knowledge regarding the innate immunomodulatory effects of vitamin D in the context of exposure to common paediatric respiratory pathogens. A relatively small number of in vitro studies were identified by our search (*n* = 11). Despite some heterogeneity in the findings, vitamin D was shown to consistently dampen the inflammatory response to common paediatric bacterial and viral pathogens with potential effects on pathogen clearance (see Section 4. Summary of findings). However, the relationship between vitamin D and the innate immune responses to respiratory pathogens is complex and the net effect will depend on the cells that respond and the response necessary for resolution of infection. Additionally, while the studies in this review clearly show that vitamin D modulates the immune response to paediatric respiratory pathogens the impact of form, dose and time of supplementation are difficult to interpret. Most of the studies only investigated one metabolite [66,69,70,71,72,73,74,75,76] and while those investigating both did report differential effects on cytokine responses the doses were variable [67], and conclusions not easily drawn. Some of the studies found a dose-dependent response to vitamin D [74], but again the interpretation was challenging because doses were not representative of normal physiological concentrations (particularly 1,25(OH)_2_D_3_) and the timing of supplementation was dictated mostly by experimental design [66,68,69,75,76]. Ultimately, improved knowledge is necessary in many areas (see Section 7. Outstanding questions). Further investigation is required to characterise the in vitro modulation of PRR expression which mediate pathogen recognition and the downstream AMP production within innate cell subsets. Additional whole live pneumococcal challenge studies are required to better depict real infection, rather than those using heat killed *S. pnemoniae* or pneumococcal cell wall fragments. Furthermore, it will be important to understand why the reduced expression of anti-viral mediators and increased autophagic flux has no apparent net effect on viral clearance (RSV and influenza virus, respectively).

Whether the in vitro findings reported in this review can be translated into a substantial in vivo benefit remains uncertain. Importantly, none of the studies utilised immune cells derived from children limiting interpretation in a paediatric context. Needless to say, despite the logistical difficulties, in vivo proof of concept within paediatric high-risk populations is necessary to gain a better representation of the childhood immune responses. Future studies should begin to focus on the in vivo immunomodulatory role of vitamin D by monitoring immune cell subset frequencies and using whole blood challenge experiments in children with known serum vitamin D levels or during vitamin D intervention trials.

## 6. Future Perspectives

Emerging research is moving towards large intervention studies examining the role of vitamin D supplementation to prevent paediatric ALRIs. For example, our “D-kids” clinical trial (ACTRN12618001174279) investigating whether vitamin D supplementation (during pregnancy and infancy) reduces the incidence of ALRIs during the first 12 months of life. Further, given the rise in the Coronavirus disease 2019 (COVID-19) pandemic and the high proportion of disease seen among those with vitamin D deficiency [108] it could be proposed that vitamin D supplementation may reduce the risk and severity of SARS-CoV-2 infection in adults [109,110].

To guide future recommendations for the use of vitamin D as an intervention against ALRIs, mechanistic research focusing on improving our understanding of the context-specific immunomodulatory activity of vitamin D must be conducted synergistically with novel clinical trials and population-based surveillance. Despite the promising experimental data indicating the immunomodulatory effects of vitamin D, population and individual level genetic variation in endogenous vitamin D utilisation must also be considered [111]. We speculate that optimal immune resilience against these respiratory pathogens is likely to require both prior and ongoing vitamin D sufficiency throughout infection since vitamin D is shown to wane during acute illness [112]. Maintaining sufficient vitamin D during infection may help reduce pathogen induced immunopathology. The findings presented in this review advocate for widespread monitoring and supplementation with vitamin D where necessary, to reduce deficiency and optimise innate immune responses to common respiratory pathogens. The cellular and molecular pathways involved in the modulation of the innate immune response by vitamin D may have profound implications for the strategic use of supplementation in reducing ALRIs in children and adults alike.

## 7. Outstanding Questions

What are the dominant DC phenotypes during respiratory bacterial and viral challenges in the presence of vitamin D? Is vitamin D involved in DC-mediated immune tolerance in response to respiratory pathogens?

Why does vitamin D promote a predominantly anti-inflammatory cytokine profile in response to S. pneumoniae yet has limited impact on the cytokine response to RSV and are these responses selectively modulated by vitamin D? Is the common view of vitamin D as an anti-inflammatory agent an oversimplification?

What is the significance of vitamin D mediated PRR modulation upon ALRI pathogen challenge? Does the altered PRR expression profile directly modulate downstream anti-microbial functions (AMP)? 

Why are the anti-viral effects (RSV and influenza) of vitamin D not associated with reduced viral load and replication? 

Do the in vitro findings highlighted in this review translate into in vivo benefits? Can the findings from adult immune cells/cell lines provide an accurate representation of the paediatric immune response?

## Figures and Tables

**Table 1:** Characteristics of studies describing the immunomodulatory effects of vitamin D on innate immune cells following challenge with *Streptococcus pneumoniae*, respiratory syncytial virus and influenza virus.

Study	Cell Source	Cell Type	Vitamin D Form, Dose and Treatment Time	Stimulation Method	Significant Innate Immune Response Change	Interpretation of Net Effects
***S. pneumoniae***						
Olliver et al., 2013 [66]	Healthy adult volunteers	Monocyte-derived DCs	1,25(OH)_2_D_3_ (100 nmol/L) pre-treatment for 24 h or at time of stimulation	*S. pneumoniae* strain T4 (encapsulated) or T4R (unencapsulated) of serotype 4 (MOI, 1 or 50) or Pneumococcal PGN (1μg/mL) or MDP (5μg/mL) for 24 h	↑ CD86 ↑ CCR7 ↓ Uptake of T4R ↑ TLR2 and NOD2 → TLR4 ↑ hBD-3 ↑ IL-1β	↑ DC maturation and migration ↓ Phagocytosis ↑ Pathogen sensing ↑ Anti-bacterial capacity ↑ Initiation of inflammation
Hoe et al., 2016 [67]	Healthy adult volunteers	PBMCs and CD14^+^ monocytes	1,25(OH)_2_D_3_ (100 nmol/L) or 25(OH)D_3_ (500nmol/L) pre-treatment for 4 h	Heat killed *S. pneumoniae* of serotype 19F (MOI, 50) for 24 h	PBMC: ↓ TNF-α, IFN-ɣ, IL-1β and IL-8 → IL-10 CD14^+^ monocytes: ↓ TNF-α ↑ IL-10 → IFN-ɣ, IL-1β and IL-8	↓ Pro-inflammatory cytokines ↑ Anti-inflammatory cytokines (in monocytes)
Subramanian et al., 2017 [68]	Healthy adult volunteers	CD66b^+^ and CD16^+^ neutrophils	1,25(OH)_2_D_3_ (100 nmol/L) or 25(OH)D_3_ (100 nmol/L) pre-treatment for 2 h or at time of stimulation	*S. pneumoniae* strain T4 or T4R of serotype 4 (MOI, 0.1) for 1 or 4 h or PGN (10μg/mL) for 4 or 6 h	↑ HNP1-3 and LL-37 ↑ TLR2 and NOD2 ↓ IL-6, IL-8 and IL-12p70 ↑ IL-4 ↑ SOCS-1 and SOCS-3 ↓ TRAF6 and NFκB	↑ Anti-bacterial capacity ↑ Pathogen sensing ↓ Pro-inflammatory cytokines ↓ Apoptosis (via ↑ IL-4) ↓ Inflammation
Anderson et al., 2020 [69]	Healthy adult volunteers	PBMCs and CD14^+^ monocytes	1,25(OH)_2_D_3_ (100 nmol/L) pre-treatment for 24 h (PBMC) or at time of stimulation (CD14^+^ monocytes)	Pneumococcal WCA (1μg/mL) for 5 days	PBMC: ↑ IL-1β and IL-10 ↓ IFN-ɣ and CD14 CD14^+^ monocytes: ↓ TLR2	↑ Initiation of inflammation ↑ Anti-inflammatory cytokines ↓ Pathogen sensing
**RSV**						
Hansdottir et al., 2008 [70]	University of Iowa Cell and Tissue Core	Human tracheobronchial epithelial cells	25(OH)D_3_ (1000 nmol/L) at time of stimulation	RSV strain A-2 (MOI, 1) for 24 h	↑ Cathelicidin mRNA	↑ Anti-viral capacity
Hansdottir et al., 2010 [71]	University of Iowa Cell and Tissue Core	Human tracheobronchial epithelial cells	1,25(OH)_2_D_3_ (1000 nmol/L) pre-treatment for 16-18 h	RSV strain A-2 (MOI, 1-2) for 24 h	↑ IκBα ↓ pSTAT1 ↓ IFN-β and CXCL10 ↓ MxA and ISG15 → Viral quantity and replication	↓ Anti-viral response ↓ Inflammation → Viral clearance
Stoppelenburg et al., 2014 [72]	A549 lung adenocarcinoma cell line	Human alveolar epithelial cells	1,25(OH)_2_D_3_ (100 nmol/L) at time of stimulation	RSV strain A-2 (MOI, 1) for 24 h	↑ IκBα ↓ pSTAT1 ↓ IFN-β ↓ IRF1 and IRF7 → Viral replication	↓ Antiviral response ↓ Inflammation → Viral clearance
Fitch et al., 2016 [73]	Healthy adult volunteers	PBMCs	1,25(OH)_2_D_3_ (10 or 100 nmol/L) at time of stimulation	RSV strain Long (10^4.9^ median tissue infectious dose/mL) for 24 h	→ CCL2, CCL8, CCL5 and IL-10	→ No change to inflammatory response
Telcian et al., 2017 [74]	BEAS-2B cell line	Human bronchial epithelial cells	1,25(OH)_2_D_3_ (10, 100 or 1000 nmol/L) pre-treatment for 16 h	RSV strain A-2 (MOI, 1) for 24 h	↑ Cathelicidin mRNA	↑ Anti-viral capacity
Anderson et al., 2020 [69]	Healthy adult volunteers	PBMCs and CD14^+^ monocytes and CD3^+^CD56^+^ NK cells	1,25(OH)_2_D_3_ (100 nmol/L) at time of stimulation	RSV strain A-2 (MOI, 1) for 24 h	PBMC: ↓ IL-6, ↓ CD14 and TLR2 CD14^+^ monocytes: ↑ TLR7 ↓ TLR2 CD3^+^CD56^+^ NK cells: ↑ TLR4	↓ Inflammation ↑↓ Pathogen sensing
**Influenza virus**						
Khare et al., 2013 [75]	A549 lung adenocarcinoma cell line	Human alveolar epithelial cells	1,25(OH)_2_D_3_ (100 nmol/L pre or 30 nmol/L post infection) for 16 h pre-treatment or 1 h post-treatment	H1N1 (1/64 HA unit) for 48 h	→ Viral quantity and cell viability pre and post ↓ Beclin-1 and Sub G1 peak pre and post treatment ↓ IL-6, TNF-α, IL-1β, RANTES and IL-8 pre and post treatment ↓ IFNβ and ISG15 pre-treatment ↑ IFNβ and ISG15 post-treatment	→ Viral clearance ↓ Autophagy ↓ Apoptosis ↓ Inflammation
Godbole et al., 2020 [76]	A549 lung adenocarcinoma cell line	Human alveolar epithelial cells	1,25(OH)_2_D_3_ (100 nmol/L) pre-treatment for 6 h plus 72 h post-infection	InfluenzaA/California/7/2009 H1N1 (MOI, 0.1) for 72 h	→ Viral quantity ↓ LC3B-Ⅱ and p62 ↑ STX17 and ATP6VOA2 ↓ Cleaved Caspase-3	→ Viral clearance ↑Autophagic flux ↓ Apoptosis

1,25(OH)_2_D_3_: 1,25 hydroxy-vitamin D3; 25(OH)D_3_: 25-hydroxyvitamin D3; DC: Dendritic cell; CCL: C-C chemokine ligand; CCR: C-C chemokine receptor; CD: Cluster of differentiation; CXCL: C-X-C chemokine ligand; hBD-3: Human beta defensin 3; MxA: human myxovirus resistance A; HNP 1-3: Human neutrophil peptide 1-3; IκBα: Inhibitor of kappa B alpha; IFN: Interferon; IRF: Interferon regulatory factor; ISG15: Interferon-stimulated protein of 15kDa; IL: Interleukin; MOI: Multiplicity of infection; MDP: Muramyl dipeptide; NK: Natural killer; NFκB: Nuclear factor kappa B; NOD: Nucleotide-binding oligomerisation domain; PGN: Peptidoglycan; PBMC: Peripheral blood mononuclear cells; RANTES: Regulated on Activation, Normal T cell Expressed and Secreted; RSV: Respiratory syncytial virus; STAT: Signal transducer and activator of transcription; *S. pneumoniae*: Streptococcus pneumoniae; SOCS: Suppressor of cytokine signalling; TLR: Toll-like receptor; TRAF: Tumour necrosis factor receptor-associated factor; TNF: Tumour necrosis factor; VDR: Vitamin D Receptor; WCA: Whole cell antigen. ↑/↓ Indicates statistically significant increase or decrease by vitamin D compared to stimulation in the absence of vitamin D. → Indicates no significant difference/change by vitamin D compared to stimulation in the absence of vitamin D.

## Data Availability

No new data were created or analyzed in this study. Data sharing is not applicable to this article.

## References

[1] Naghavi M., Abajobir A.A., Abbafati C., Abbas K.M., Abd-Allah F., Abera S.F., Aboyans V., Adetokunboh O., Afshin A., Agrawal A. (2017). Global, regional, and national age-sex specific mortality for 264 causes of death, 1980–2016: A systematic analysis for the Global Burden of Disease Study 2016. Lancet.

[2] Binks M.J., Beissbarth J., Oguoma V.M., Pizzutto S.J., Leach A.J., Smith-Vaughan H.C., McHugh L., Andrews R.M., Webby R., Morris P.S. (2020). Acute lower respiratory infections in Indigenous infants in Australia’s Northern Territory across three eras of pneumococcal conjugate vaccine use (2006–15): A population-based cohort study. Lancet Child Adolesc. Health.

[3] Tennant P.W.G., Gibson G.J., Parker L., Pearce M., Msc P.W.G.T. (2010). Childhood Respiratory Illness and Lung Function at Ages 14 and 50 Years. Chest.

[4] A McAllister D., Liu L., Shi T., Chu Y., Reed C., Burrows J., Adeloye D., Rudan I., E Black R., Campbell H. (2019). Global, regional, and national estimates of pneumonia morbidity and mortality in children younger than 5 years between 2000 and 2015: A systematic analysis. Lancet Glob. Health.

[5] Histoshi T., McAllister D.A., O’Brien K.L., Simoes E.A.F., Madhi S.A., Gessner B.D., Polack F.P., Balsells E., Acacio S., Aguayo C. (2017). Global, regional, and national disease burden estimates of acute lower respiratory infections due to respiratory syncytial virus in young children in 2015: A systematic review and modelling study. Lancet.

[6] Nair H., Brooks W.A., Katz M., Roca A., A Berkley J., Madhi S.A., Simmerman J.M., Gordon A., Sato M., Howie S. (2011). Global burden of respiratory infections due to seasonal influenza in young children: A systematic review and meta-analysis. Lancet.

[7] Dawood F.S., Ricks P., Njie G.J., Daugherty M., Davis W., A Fuller J., Winstead A., McCarron M., Scott L.C., Chen D. (2020). Observations of the global epidemiology of COVID-19 from the prepandemic period using web-based surveillance: A cross-sectional analysis. Lancet Infect. Dis..

[8] Alderson M. (2016). Status of research and development of pediatric vaccines for Streptococcus pneumoniae. Vaccine.

[9] Jefferson T., Rivetti A., Di Pietrantonj C., Demicheli V. (2018). Vaccines for preventing influenza in healthy children. Cochrane Database Syst. Rev..

[10] Licciardi P.V., Papadatou I. (2019). Pneumococcal Vaccines: Challenges and Prospects. Vaccines.

[11] Hare K., Smith-Vaughan H., Chang A.B., Pizzutto S., Petsky H.L., Bn G.B.M., Leach A.J. (2017). Propensity of pneumococcal carriage serotypes to infect the lower airways of children with chronic endobronchial infections. Vaccine.

[12] Mameli C., Cocchi I., Fumagalli M., Zuccotti G. (2019). Influenza Vaccination: Effectiveness, Indications, and Limits in the Pediatric Population. Front. Pediatr..

[13] I Mazur N., Higgins D., Nunes M.C., Melero J.A., Langedijk A.C., Horsley N., Buchholz U.J., Openshaw P.J., McLellan J.S., A Englund J. (2018). The respiratory syncytial virus vaccine landscape: Lessons from the graveyard and promising candidates. Lancet Infect. Dis..

[14] Ramasamy M.N., Minassian A.M., Ewer K.J., Flaxman A.L., Folegatti P.M., Owens D.R., Voysey M., Aley P.K., Angus B., Babbage G. (2020). Safety and immunogenicity of ChAdOx1 nCoV-19 vaccine administered in a prime-boost regimen in young and old adults (COV002): A single-blind, randomised, controlled, phase 2/3 trial. Lancet.

[15] Walsh E.E., Frenck R.W., Falsey A.R., Kitchin N., Absalon J., Gurtman A., Lockhart S., Neuzil K., Mulligan M.J., Bailey R. (2020). Safety and Immunogenicity of Two RNA-Based Covid-19 Vaccine Candidates. N. Engl. J. Med..

[16] Cashman K.D. (2020). Vitamin D Deficiency: Defining, Prevalence, Causes, and Strategies of Addressing. Calcif. Tissue Int..

[17] Cashman K.D., Dowling K.G., Škrabáková Z., Gonzalez-Gross M., Valtueña J., De Henauw S., Moreno L., Damsgaard C.T., Michaelsen K.F., Mølgaard C. (2016). Vitamin D deficiency in Europe: Pandemic?. Am. J. Clin. Nutr..

[18] Hilger J., Friedel A., Herr R., Rausch T., Roos F., Wahl D.A., Pierroz D.D., Weber P., Hoffmann K. (2014). A systematic review of vitamin D status in populations worldwide. Br. J. Nutr..

[19] Binks M.J., Smith-Vaughan H.C., Marsh R., Chang A.B., Andrews R.M. (2016). Cord blood vitamin D and the risk of acute lower respiratory infection in Indigenous infants in the Northern Territory. Med J. Aust..

[20] Grant C.C., Stewart A.W., Scragg R., Milne T., Rowden J., Ekeroma A., Wall C., Mitchell E.A., Crengle S., Trenholme A. (2013). Vitamin D During Pregnancy and Infancy and Infant Serum 25-Hydroxyvitamin D Concentration. Pediatrics.

[21] Szodoray P., Nakken B., Gaal J., Jonsson R., Szegedi A., Zold E., Szegedi G., Brun J.G., Gesztelyi R., Zeher M. (2008). The Complex Role of Vitamin D in Autoimmune Diseases. Scand. J. Immunol..

[22] Young M.R.I., Xiong Y. (2018). Influence of vitamin D on cancer risk and treatment: Why the variability?. Trends Cancer Res..

[23] Agrawal D.K., Yin K. (2014). Vitamin D and inflammatory diseases. J. Inflamm. Res..

[24] Moukarzel S., Ozias M.K., Kerling E.H., Christifano D.N., Wick J.A., Colombo J., Carlson S.E. (2018). Maternal Vitamin D Status and Infant Infection. Nutrients.

[25] Roth D.E., Shah R., Black R.E., Baqui A.H. (2010). Vitamin D status and acute lower respiratory infection in early childhood in Sylhet, Bangladesh. Acta Paediatr..

[26] Wayse V., Yousafzai A.K., Mogale K., Filteau S. (2004). Association of subclinical vitamin D deficiency with severe acute lower respiratory infection in Indian children under 5 y. Eur. J. Clin. Nutr..

[27] Karatekin G., Kaya A., Salihoğlu O., Balci H., Nuhoğlu A. (2007). Association of subclinical vitamin D deficiency in newborns with acute lower respiratory infection and their mothers. Eur. J. Clin. Nutr..

[28] Belderbos M.E., Houben M.L., Wilbrink B., Lentjes E., Bloemen E.M., Kimpen J.L.L., Rovers M., Bont L. (2011). Cord Blood Vitamin D Deficiency Is Associated With Respiratory Syncytial Virus Bronchiolitis. Pediatrics.

[29] Shin Y.H., Yu J., Kim K.W., Ahn K., Hong S.A., Lee E., Yang S.-I., Jung Y.-H., Kim H.Y., Seo J.-H. (2013). Association between cord blood 25-hydroxyvitamin D concentrations and respiratory tract infections in the first 6 months of age in a Korean population: A birth cohort study (COCOA). Korean J. Pediatr..

[30] Jat K.R. (2016). Vitamin D deficiency and lower respiratory tract infections in children: A systematic review and meta-analysis of observational studies. Trop. Dr..

[31] Montecino M., Stein G.S., Stein J.L., Lian J.B., Van Wijnen A.J., Carvallo L., Marcellini S., Cruzat V., Arriagada G. (2008). Vitamin D control of gene expression: Temporal and spatial parameters for organization of the regulatory machinery. Crit. Rev. Eukaryot. Gene Expr..

[32] Ramagopalan S.V., Heger A., Berlanga A.J., Maugeri N.J., Lincoln M.R., Burrell A., Handunnetthi L., Handel A.E., Disanto G., Orton S.-M. (2010). A ChIP-seq defined genome-wide map of vitamin D receptor binding: Associations with disease and evolution. Genome Res..

[33] Zhang X., Ho S.-M. (2011). Epigenetics meets endocrinology. J. Mol. Endocrinol..

[34] Zhang D.E., Hetherington C.J., A Gonzalez D., Chen H.M., Tenen D.G. (1994). Regulation of CD14 expression during monocytic differentiation induced with 1 alpha,25-dihydroxyvitamin D3. J. Immunol..

[35] Arababadi M.K., Nosratabadi R., Asadikaram G. (2018). Vitamin D and toll like receptors. Life Sci..

[36] Cantorna M.T. (2010). Mechanisms underlying the effect of vitamin D on the immune system. Proc. Nutr. Soc..

[37] Hossein-Nezhad A., Spira A., Holick M. (2013). Influence of Vitamin D Status and Vitamin D3 Supplementation on Genome Wide Expression of White Blood Cells: A Randomized Double-Blind Clinical Trial. PLoS ONE.

[38] Nurminen V., Seuter S., Carlberg C. (2019). Primary Vitamin D Target Genes of Human Monocytes. Front. Physiol..

[39] Calton E.K., Keane K.N., Newsholme P., Soares M.J. (2015). The Impact of Vitamin D Levels on Inflammatory Status: A Systematic Review of Immune Cell Studies. PLoS ONE.

[40] Wang J.W., Hogan P.G., Hunstad D.A., Fritz S.A. (2015). Vitamin D Sufficiency and Staphylococcus Aureus Infection in Children. Pediatr. Infect. Dis. J..

[41] Thomason J., Rentsch C.T., Stenehjem E., Botero A.H., Rimland D. (2015). Association between vitamin D deficiency and methicillin-resistant Staphylococcus aureus infection. Infection.

[42] Nouari W., Ysmail-Dahlouk L., Aribi M. (2016). Vitamin D3 enhances bactericidal activity of macrophage against Pseudomonas aeruginosa. Int. Immunopharmacol..

[43] Martineau A.R., Jolliffe D.A., Hooper R.L., Greenberg L., Aloia J.F., Bergman P., Dubnov-Raz G., Esposito S., Ganmaa D., Ginde A.A. (2017). Vitamin D supplementation to prevent acute respiratory tract infections: Systematic review and meta-analysis of individual participant data. BMJ.

[44] Martineau A.R., Jolliffe D.A., Greenberg L., Aloia J.F., Bergman P., Dubnov-Raz G., Esposito S., Ganmaa D., Ginde A.A., Goodall E.C. (2019). Vitamin D supplementation to prevent acute respiratory infections: Individual participant data meta-analysis. Health Technol. Assess..

[45] Holick M.F., Feldman D., Pike J.W., Adams J.S. (2011). Chapter 2—Photobiology of Vitamin D. Vitamin D (Third Edition).

[46] Bikle D. (2020). Vitamin D: Newer Concepts of Its Metabolism and Function at the Basic and Clinical Level. J. Endocr. Soc..

[47] Passeron T., Bouillon R., Callender V., Cestari T.F., Diepgen T., Green A.C., Van Der Pols J.C., Bernard B., Ly F., Bernerd F. (2019). Sunscreen photoprotection and vitamin D status. Br. J. Dermatol..

[48] Holick M. (2009). Vitamin D Status: Measurement, Interpretation, and Clinical Application. Ann. Epidemiol..

[49] Liu P.T., Stenger S., Li H., Wenzel L., Tan B.H., Krutzik S.R., Ochoa M.T., Schauber J., Wu K., Meinken C. (2006). Toll-Like Receptor Triggering of a Vitamin D-Mediated Human Antimicrobial Response. Science.

[50] Mann E., Chambers E.S., Pfeffer P.E., Hawrylowicz C.M. (2014). Immunoregulatory mechanisms of vitamin D relevant to respiratory health and asthma. Ann. New York Acad. Sci..

[51] Haussler M.R., Jurutka P.W., Mizwicki M., Norman A.W. (2011). Vitamin D receptor (VDR)-mediated actions of 1α,25(OH)2vitamin D3: Genomic and non-genomic mechanisms. Best Pract. Res. Clin. Endocrinol. Metab..

[52] BioRender. https://biorender.com/.

[53] Bhalla A.K., Amento E.P., Clemens T.L., Holick M.F., Krane S.M. (1983). Specific high-affinity receptors for 1,25-dihydroxyvitamin D_3_ in human peripheral blood mononuclear cells: presence in monocytes and induction in T lymphocytes following activation. J. Clin. Endocrinol. Metab..

[54] McNally P., Coughlan C., Bergsson G., Doyle M., Taggart C.C., Adorini L., Uskokovic M., El Nazir B., Murphy P., Greally P. (2011). Vitamin D receptor agonists inhibit pro-inflammatory cytokine production from the respiratory epithelium in cystic fibrosis. J. Cyst. Fibros..

[55] Takahashi K., Nakayama Y., Horiuchi H., Ohta T., Komoriya K., Ohmori H., Kamimura T. (2002). Human neutrophils express messenger RNA of vitamin d receptor and respond to 1α,25-dihydroxyvitamin D3. Immunopharmacol. Immunotoxicol..

[56] Brennan A., Katz D.R., Nunn J.D., Barker S., Hewison M., Fraher L.J., O’Riordan J.L. (1987). Dendritic cells from human tissues express receptors for the immunoregulatory vitamin D3 metabolite, dihydroxycholecalciferol. Immunology.

[57] Pike J.W., Meyer M.B. (2010). The Vitamin D Receptor: New Paradigms for the Regulation of Gene Expression by 1,25-Dihydroxyvitamin D3. Endocrinol. Metab. Clin. North Am..

[58] Jeon S.-M., Shin E.-A. (2018). Exploring vitamin D metabolism and function in cancer. Exp. Mol. Med..

[59] Wang T.-T., Nestel F.P., Bourdeau V., Nagai Y., Wang Q., Liao J., Tavera-Mendoza L., Lin R., Hanrahan J.W., Mader S. (2004). Cutting Edge: 1,25-Dihydroxyvitamin D3 Is a Direct Inducer of Antimicrobial Peptide Gene Expression. J. Immunol..

[60] Gombart A.F., Borregaard N., Koeffler H.P. (2005). Human cathelicidin antimicrobial peptide (CAMP) gene is a direct target of the vitamin D receptor and is strongly up-regulated in myeloid cells by 1,25-dihydroxyvitamin D 3. FASEB J..

[61] Kahlenberg J.M., Kaplan M.J. (2013). Little Peptide, Big Effects: The Role of LL-37 in Inflammation and Autoimmune Disease. J. Immunol..

[62] Greiller C.L., Martineau A.R. (2015). Modulation of the Immune Response to Respiratory Viruses by Vitamin D. Nutrients.

[63] Lang P.O., Aspinall R. (2017). Vitamin D Status and the Host Resistance to Infections: What It Is Currently (Not) Understood. Clin. Ther..

[64] Sassi F., Tamone C., D’Amelio P. (2018). Vitamin D: Nutrient, Hormone, and Immunomodulator. Nutrients.

[65] Zdrenghea M., Makrinioti H., Bagacean C., Bush A., Johnston S.L., Stanciu L.A. (2017). Vitamin D modulation of innate immune responses to respiratory viral infections. Rev. Med. Virol..

[66] Olliver M., Spelmink L., Hiew J., Meyer-Hoffert U., Henriques-Normark B., Bergman P. (2013). Immunomodulatory Effects of Vitamin D on Innate and Adaptive Immune Responses to Streptococcus pneumoniae. J. Infect. Dis..

[67] Hoe E., Nathanielsz J., Toh Z.Q., Spry L., Marimla R., Balloch A., Mulholland K., Licciardi P.V. (2016). Anti-Inflammatory Effects of Vitamin D on Human Immune Cells in the Context of Bacterial Infection. Nutrients.

[68] Subramanian K., Bergman P., Henriques-Normark B. (2017). Vitamin D Promotes Pneumococcal Killing and Modulates Inflammatory Responses in Primary Human Neutrophils. J. Innate Immun..

[69] Anderson J., Do L.A.H., Toh Z.Q., Hoe E., Reitsma A., Mulholland K., Licciardi P.V. (2020). Vitamin D Induces Differential Effects on Inflammatory Responses During Bacterial and/or Viral Stimulation of Human Peripheral Blood Mononuclear Cells. Front. Immunol..

[70] Hansdottir S., Monick M.M., Hinde S.L., Lovan N., Look D.C., Hunninghake G.W. (2008). Respiratory Epithelial Cells Convert Inactive Vitamin D to Its Active Form: Potential Effects on Host Defense. J. Immunol..

[71] Hansdottir S., Monick M.M., Lovan N., Powers L.S., Gerke A.K., Hunninghake G.W. (2009). Vitamin D Decreases Respiratory Syncytial Virus Induction of NF-κB–Linked Chemokines and Cytokines in Airway Epithelium While Maintaining the Antiviral State. J. Immunol..

[72] Stoppelenburg A.J., Von Hegedus J.H., Veld R.H.I., Bont L., Boes M. (2013). Defective control of vitamin D receptor-mediated epithelial STAT1 signalling predisposes to severe respiratory syncytial virus bronchiolitis. J. Pathol..

[73] Fitch N., Becker A.B., HayGlass K.T. (2016). Vitamin D [1,25(OH)2D3] Differentially Regulates Human Innate Cytokine Responses to Bacterial versus Viral Pattern Recognition Receptor Stimuli. J. Immunol..

[74] Telcian A.G., Zdrenghea M., Edwards M.R., Laza-Stanca V., Mallia P., Johnston S.L., Stanciu L.A. (2017). Vitamin D increases the antiviral activity of bronchial epithelial cells in vitro. Antivir. Res..

[75] Khare D., Godbole N.M., Pawar S.D., Mohan V., Pandey G., Gupta S., Kumar D., Dhole T.N., Godbole M.M. (2013). Calcitriol [1, 25[OH]2 D3] pre- and post-treatment suppresses inflammatory response to influenza A (H1N1) infection in human lung A549 epithelial cells. Eur. J. Nutr..

[76] Godbole N.M., Sinha R.A., Tiwari S., Pawar S.D., Dhole T.N. (2020). Analysis of influenza virus-induced perturbation in autophagic flux and its modulation during Vitamin D3 mediated anti-apoptotic signaling. Virus Res..

[77] Wahl B., O’Brien K.L., Greenbaum A., Majumder A., Liu L., Chu Y., Lukšić I., Nair H., McAllister D.A., Campbell H. (2018). Burden of Streptococcus pneumoniae and Haemophilus influenzae type b disease in children in the era of conjugate vaccines: Global, regional, and national estimates for 2000–15. Lancet Glob. Health.

[78] Troeger C., Blacker B., A Khalil I., Rao P.C., Cao J., Zimsen S.R.M., Albertson S.B., Deshpande A., Farag T., Abebe Z. (2018). Estimates of the global, regional, and national morbidity, mortality, and aetiologies of lower respiratory infections in 195 countries, 1990–2016: A systematic analysis for the Global Burden of Disease Study 2016. Lancet Infect. Dis..

[79] Pletz M., Terkamp C., Schumacher U., Rohde G., Schütte H., Welte T., Bals R., CAPNETZ-Study Group (2014). Vitamin D deficiency in community-acquired pneumonia: Low levels of 1,25(OH)2 D are associated with disease severity. Respir. Res..

[80] Hartl D., Tirouvanziam R., Laval J., Greene C.M., Habiel D., Sharma L., Yildirim A.Ö., Cruz C.S.D., Hogaboam C.M. (2018). Innate Immunity of the Lung: From Basic Mechanisms to Translational Medicine. J. Innate Immun..

[81] Sadeghi K., Wessner B., Laggner U., Ploder M., Tamandl D., Friedl J., Zügel U., Steinmeyer A., Pollak A., Roth E. (2006). Vitamin D3 down-regulates monocyte TLR expression and triggers hyporesponsiveness to pathogen-associated molecular patterns. Eur. J. Immunol..

[82] Wang T.-T., Dabbas B., Laperriere D., Bitton A.J., Soualhine H., Tavera-Mendoza L.E., Dionne S., Servant M.J., Bitton A., Seidman E.G. (2010). Direct and Indirect Induction by 1,25-Dihydroxyvitamin D3of the NOD2/CARD15-Defensin β2 Innate Immune Pathway Defective in Crohn Disease. J. Biol. Chem..

[83] Bartels L.E., Hvas C.L., Agnholt J., Dahlerup J.F., Agger R. (2010). Human dendritic cell antigen presentation and chemotaxis are inhibited by intrinsic 25-hydroxy vitamin D activation. Int. Immunopharmacol..

[84] Griffin M.D., Lutz W., Phan V.A., Bachman L.A., Mckean D.J., Kumar R. (2001). Dendritic cell modulation by 1,25 dihydroxyvitamin D3 and its analogs: A vitamin D receptor-dependent pathway that promotes a persistent state of immaturity in vitro and in vivo. Proc. Natl. Acad. Sci. USA.

[85] Penna G., Adorini L. (2000). 1α,25-Dihydroxyvitamin D3Inhibits Differentiation, Maturation, Activation, and Survival of Dendritic Cells Leading to Impaired Alloreactive T Cell Activation. J. Immunol..

[86] Hewison M., Freeman L., Hughes S.V., Evans K.N., Bland R., Eliopoulos A.G., Kilby M.D., Moss P.A.H., Chakraverty R. (2003). Differential Regulation of Vitamin D Receptor and Its Ligand in Human Monocyte-Derived Dendritic Cells. J. Immunol..

[87] Adorini L. (2003). Tolerogenic dendritic cells induced by vitamin D receptor ligands enhance regulatory T cells inhibiting autoimmune diabetes. Ann. N. Y. Acad. Sci..

[88] Marriott H.M., Gascoyne K.A., Gowda R., Geary I., Nicklin M.J.H., Iannelli F., Pozzi G., Mitchell T.J., Whyte M.K.B., Sabroe I. (2011). Interleukin-1β Regulates CXCL8 Release and Influences Disease Outcome in Response to Streptococcus pneumoniae, Defining Intercellular Cooperation between Pulmonary Epithelial Cells and Macrophages. Infect. Immun..

[89] Verway M., Bouttier M., Wang T.-T., Carrier M., Calderon M., Manuella B., Devemy E., McIntosh F., Divangahi M., Behr M.A. (2013). Vitamin D Induces Interleukin-1β Expression: Paracrine Macrophage Epithelial Signaling Controls M. tuberculosis Infection. PLoS Pathog..

[90] Greiller C.L., Suri R., Jolliffe D.A., Kebadze T., Hirsman A.G., Griffiths C.J., Johnston S.L., Martineau A.R. (2019). Vitamin D attenuates rhinovirus-induced expression of intercellular adhesion molecule-1 (ICAM-1) and platelet-activating factor receptor (PAFR) in respiratory epithelial cells. J. Steroid Biochem. Mol. Biol..

[91] Maxwell C.S., Carbone E.T., Wood R.J. (2012). Better newborn vitamin D status lowers RSV-associated bronchiolitis in infants. Nutr. Rev..

[92] Russell C.D., Unger S.A., Walton M., Schwarze J. (2017). The Human Immune Response to Respiratory Syncytial Virus Infection. Clin. Microbiol. Rev..

[93] Medzhitov R. (2001). Toll-like receptors and innate immunity. Nat. Rev. Immunol..

[94] Haynes L.M., Moore D.D., Kurt-Jones E.A., Finberg R.W., Anderson L.J., Tripp R.A. (2001). Involvement of Toll-Like Receptor 4 in Innate Immunity to Respiratory Syncytial Virus. J. Virol..

[95] Moretta L., Ferlazzo G., Mingari M.C., Melioli G., Moretta A. (2003). Human natural killer cell function and their interactions with dendritic cells. Vaccine.

[96] Kim T.H., Lee H.K. (2014). Innate immune recognition of respiratory syncytial virus infection. BMB Rep..

[97] Janssen R., Bont L., Siezen C.L.E., Hodemaekers H.M., Ermers M.J., Doornbos G., Slot R.V.T., Wijmenga C., Goeman J.J., Kimpen J.L.L. (2007). Genetic Susceptibility to Respiratory Syncytial Virus Bronchiolitis Is Predominantly Associated with Innate Immune Genes. J. Infect. Dis..

[98] McNally J.D., Sampson M., Matheson L.A., Hutton B., Little J. (2013). Vitamin D receptor (VDR) polymorphisms and severe RSV bronchiolitis: A systematic review and meta-analysis. Pediatr. Pulmonol..

[99] Harcourt J.L., McDonald M., Svoboda P., Pohl J., Tatti K.M., Haynes L.M. (2016). Human cathelicidin, LL-37, inhibits respiratory syncytial virus infection in polarized airway epithelial cells. BMC Res. Notes.

[100] Schögler A., Muster R.J., Kieninger E., Casaulta C., Tapparel C., Jung A., Moeller A., Geiser T., Regamey N., Alves M.P. (2015). Vitamin D represses rhinovirus replication in cystic fibrosis cells by inducing LL-37. Eur. Respir. J..

[101] Berry D.J., Hesketh K., Power C., Hyppönen E. (2011). Vitamin D status has a linear association with seasonal infections and lung function in British adults. Br. J. Nutr..

[102] Urashima M., Mezawa H., Noya M., Camargo C.A. (2014). Effects of vitamin D supplements on influenza A illness during the 2009 H1N1 pandemic: A randomized controlled trial. Food Funct..

[103] Chen X., Liu S., Goraya M.U., Maarouf M., Huang S., Chen J.-L. (2018). Host Immune Response to Influenza A Virus Infection. Front. Immunol..

[104] Mariño G., Niso-Santano M., Baehrecke E.H., Kroemer G. (2014). Self-consumption: The interplay of autophagy and apoptosis. Nat. Rev. Mol. Cell Biol..

[105] Yoshii S.R., Mizushima N. (2017). Monitoring and Measuring Autophagy. Int. J. Mol. Sci..

[106] Yuk J.-M., Shin D.-M., Lee H.-M., Yang C.-S., Jin H.S., Kim K.-K., Lee Z.-W., Lee S.H., Kim J.-M., Jo E.-K. (2009). Vitamin D3 Induces Autophagy in Human Monocytes/Macrophages via Cathelicidin. Cell Host Microbe.

[107] Tripathi S., Tecle T., Verma A., Crouch E., White M., Hartshorn K.L. (2013). The human cathelicidin LL-37 inhibits influenza A viruses through a mechanism distinct from that of surfactant protein D or defensins. J. Gen. Virol..

[108] Mitchell F. (2020). Vitamin-D and COVID-19: Do deficient risk a poorer outcome?. Lancet Diabetes Endocrinol..

[109] Martineau A.R., Forouhi N.G. (2020). Vitamin D for COVID-19: A case to answer?. Lancet Diabetes Endocrinol..

[110] Meltzer D.O., Best T.J., Zhang H., Vokes T., Arora V., Solway J. (2020). Association of Vitamin D Status and Other Clinical Characteristics With COVID-19 Test Results. JAMA Netw. Open.

[111] Hossein-Nezhad A., Kalajian T.A., Song A., Holick M. (2019). Disassociation of Vitamin D’s Calcemic Activity and Non-calcemic Genomic Activity and Individual Responsiveness: A Randomized Controlled Double-Blind Clinical Trial. Sci. Rep..

[112] Gama R., Waldron J.L., Ashby H.L., Cornes M.P., Bechervaise J., Razavi C., Thomas O.L., Chugh S., Deshpande S., Ford C. (2012). Hypovitaminosis D and disease: Consequence rather than cause?. BMJ.

